# Public Perspectives on Decisions About Emergency Care Seeking for Care Unrelated to COVID-19 During the COVID-19 Pandemic

**DOI:** 10.1001/jamanetworkopen.2021.20940

**Published:** 2021-08-19

**Authors:** Rebecca Gale, Samuel Eberlein, Garth Fuller, Carine Khalil, Christopher V. Almario, Brennan M.R. Spiegel

**Affiliations:** 1 Cedars-Sinai Center for Outcomes Research and Education, Los Angeles, California; 2 Department of Medicine, Cedars-Sinai Medical Center, Los Angeles, California; 3 Karsh Division of Gastroenterology and Hepatology, Cedars-Sinai Medical Center, Los Angeles, California; 4 Division of Health Services Research, Cedars-Sinai Medical Center, Los Angeles, California; 5 Division of Informatics, Cedars-Sinai Medical Center, Los Angeles, California; 6 Department of Health Policy and Management, University of California, Los Angeles Fielding School of Public Health

## Abstract

**Importance:**

Delaying critical care for treatable conditions owing to fear of contracting COVID-19 in the emergency department (ED) is associated with avoidable morbidity and mortality.

**Objective:**

To assess and quantify how people decided whether to present to the ED during the COVID-19 pandemic for care unrelated to COVID-19 using conjoint analysis, a form of trade-off analysis that examines how individuals make complex decisions.

**Design, Setting, and Participants:**

This cross-sectional survey study was conducted using a nationwide sample from June 1, 2020, during the initial peak of the COVID-19 pandemic. Included participants were adults aged 18 years or older in the US who self-reported that they had not tested positive for COVID-19. Data were analyzed from July 2020 through May 2021.

**Exposures:**

Participants completed a self-administered online survey.

**Main Outcomes and Measures:**

Using a choice-based conjoint analysis survey, the relative importance was assessed for the following attributes for individuals in deciding whether to seek ED care for symptoms consistent with myocardial infarction or appendicitis: reduction in chance of dying because of ED treatment, crowdedness of ED with other patients, and chance of contracting COVID-19 in the ED. We also performed latent class analyses using conjoint data to identify distinct segments of the respondent population with similar choice patterns. Logistic regression was then used to explore whether patient sociodemographics and political affiliations were factors associated with decision-making.

**Results:**

Among 1981 individuals invited to participate, 933 respondents (47.1%) completed the survey; participants’ mean (SD) age was 40.1 (13.0) years, and 491 (52.6%) were women. In latent class analyses, 158 individuals (16.9%) with symptoms of myocardial infarction and 238 individuals (25.5%) with symptoms of appendicitis prioritized avoidance of COVID-19 exposure in the ED (ie, chance of contracting COVID-19 in the ED or crowdedness of ED with other patients) over seeking appropriate care for symptoms. Having a usual source of care was a factor associated with lower odds of prioritizing avoidance of COVID-19 exposure (myocardial infarction scenario: adjusted odds ratio, 0.49 [95% CI, 0.32-0.76]; *P* = .001; appendicitis scenario: adjusted odds ratio, 0.57 [95% CI, 0.40-0.82]; *P* = .003), but most sociodemographic factors and political affiliations were not factors associated with decision-making.

**Conclusions and Relevance:**

This study found that up to one-quarter of individuals were willing to forgo potentially life-saving ED care to avoid exposure to COVID-19. These findings suggest that health care systems and public health organizations should develop effective communications for patients and the community at large that reassure and encourage timely health care for critical needs during the ongoing COVID-19 pandemic and other scenarios.

## Key Points

**Question:**

What do people prioritize when deciding whether to present to the emergency department during the COVID-19 pandemic for care unrelated to COVID-19?

**Findings:**

In this survey study of 933 US adults, we found that 16.9% and 25.5% of individuals confronted with scenarios consistent with myocardial infarction or appendicitis, respectively, prioritized avoidance of COVID-19 exposure in the emergency department over seeking appropriate care. Sociodemographics, political affiliations, and personal knowledge, attitudes, and beliefs regarding COVID-19 were not factors associated with decision-making regarding emergency care seeking.

**Meaning:**

These findings suggest that health care systems and public health organizations should develop effective communications for patients and the community at large that reassure and encourage appropriate, timely health care for critical needs, not only during the ongoing COVID-19 pandemic, but also for future infectious outbreaks and other scenarios that could promote maladaptive pathogen-avoidance behaviors.

## Teaser

This survey study investigates individuals' decision-making process for seeking emergency department treatment for conditions unrelated to COVID-19 during the COVID-19 pandemic using hypothetical scenarios.

## Introduction

As of May 20, 2021, there have been more than 33 million cases and 588 000 deaths in the US from COVID-19,^1^ placing emergency departments (EDs) at the forefront of the pandemic. Simultaneously, hospitals have reported decreases in the number of patients presenting for stroke, acute coronary syndrome, and uncontrolled hyperglycemia, among several other critical conditions.^2,3,4,5,6,7,8,9,10,11,12,13^ For example, Oseran et al^4^ observed decreases by 29.6% and 44.7% in cardiovascular and gastroenterology admissions, respectively, from March 1 to April 30, 2020, compared with the same period in 2019.

Fear of acquiring COVID-19 in health care settings has led many people to forego emergency care despite experiencing potentially life-threatening illnesses,^2,3,4,5,6,7,8,9,10,11,12,13^ a decision thought to be associated with the behavioral immune system, a set of cognitive responses that aid in avoiding social exposure to pathogens.^14,15^ Although pathogen-avoidance behaviors are evolutionarily adaptive, when applied excessively they may lead patients to incur far greater risk by staying home compared with the actual risk of contracting an infectious disease, such as COVID-19, by seeking care. Nonetheless, studies have found that patients have avoided receiving necessary emergency care during the pandemic^2,3,4,5,6,7,8,9,10,11,12,13^ despite evidence of a low risk of contracting COVID-19 in health care settings.^16^

In light of the association of the COVID-19 pandemic with forgone medical care^2,3,4,5,6,7,8,9,10,11,12,13^ and the potential for future outbreaks of COVID-19 or other community pathogens, we aimed to quantitively and qualitatively examine the willingness of a nationwide sample of US individuals to seek emergency care during the initial peak of COVID-19. This pandemic was used as a model health care crisis with potential to be associated with maladaptive pathogen-avoidance behaviors. Moreover, to examine the trade-offs these individuals made, we used conjoint analysis, a technique that evaluates how people navigate complex decisions by balancing competing factors. Additionally, we assessed whether sociodemographic factors, political affiliations, and knowledge, attitudes, and beliefs regarding COVID-19 were factors associated with emergency care decision-making.

## Methods

### Study Design and Participant Recruitment

This survey study was approved by the Cedars-Sinai Institutional Review Board and followed the American Association for Public Opinion Research (AAPOR) reporting guideline.^17^ We performed a cross-sectional, self-administered, online survey of a nationwide sample of US adults who had not tested positive for COVID-19. The objective was to understand the trade-offs individuals made when deciding to seek care at the ED for potentially life-threatening conditions during a pandemic.

We collaborated with Cint (Stockholm, Sweden), a survey research firm, to recruit respondents. Cint drew from a sample of more than 20 million panelists in the US who signed up to receive invitations to complete surveys in exchange for Cint marketplace points, which we describe elsewhere.^18,19^ To recruit a cohort approximating a nationally representative sample, Cint used quotas for age, sex, and US region based on the latest US Census data.

On June 1, 2020, during the initial peak of the COVID-19 pandemic in the US, Cint sent an invitation email to panelists aged 18 years or older. Respondents who clicked the survey link were informed that the goal was to “learn how people make decisions about whether to go to the emergency room (ER) for treatment during the COVID-19 pandemic.”

### Study Population

The survey instrument is shown in eAppendix 1 in the Supplement, and descriptions of the myocardial infarction and appendicitis scenarios are shown in eAppendix 2 and eAppendix 3 in the Supplement. All respondents who accessed the survey were asked about their experience with COVID-19, and those who self-reported a prior positive COVID-19 diagnosis were excluded. Thus, all individuals aged 18 years or older who did not report a positive COVID-19 test result were eligible. Before proceeding with the remaining questions, all participants reviewed a study information page and provided informed consent.

### Survey Instrument

#### Conjoint Analysis to Assess Decision-Making on Emergency Care Seeking During a Pandemic

##### Overview of Conjoint Analysis

Participants completed a conjoint analysis exercise, a technique that quantifies how respondents make trade-offs during decision-making. Conjoint analysis is based on the premise that decision-making scenarios can be described by a combination of attributes and is valued based on the levels of its attributes.^20^ It is administered via an online interactive module in which respondents are presented with a series of unique, side-by-side scenarios and tasked with selecting their preferred scenario (Figure).

**Figure.**
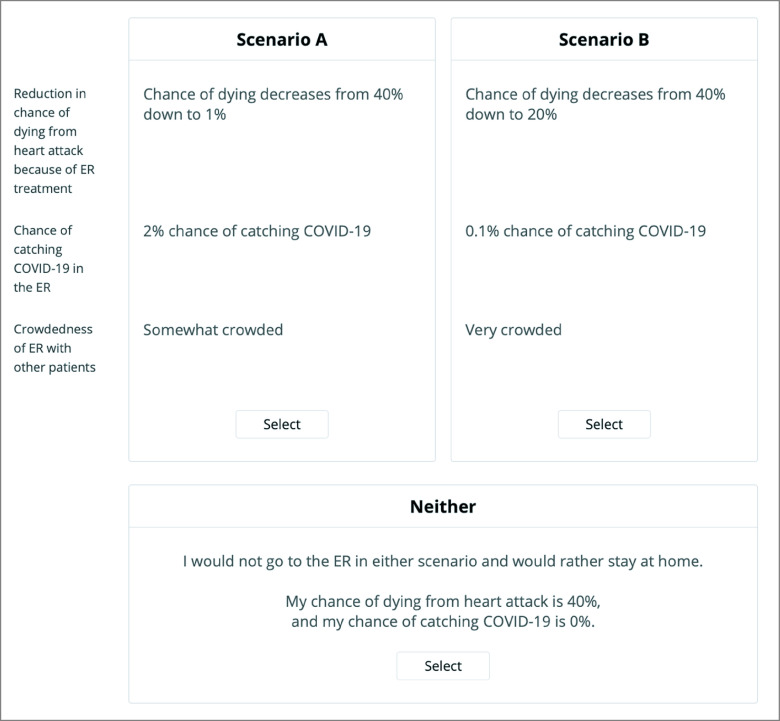
Sample Choice-Based Conjoint Exercise Participants consider 2 hypothetical scenarios side by side and decide in which situation, if any, they would be more likely to go to the emergency department for treatment. ER indicates emergency room.

##### Choice-Based Conjoint Analysis for Evaluating Decision-Making on Emergency Care Seeking

We employed choice-based conjoint (CBC) analysis using Lighthouse Studio software version 9.8.1 (Sawtooth Software) to determine how patients made decisions regarding emergency care seeking during the COVID-19 pandemic. The CBC exercises were developed following International Society for Pharmacoeconomic and Outcomes Research best practices.^20^ Table 1 displays the 3 attributes and their levels for the 2 scenarios; in 1 scenario, an individual was experiencing symptoms consistent with myocardial infarction, while the other scenario indicated an individual experiencing appendicitis. Prior to completing the CBC exercises, respondents were given information about the scenarios and attributes (eAppendices 2 and 3 in the Supplement).

**Table 1.** Attributes and Levels Included in Conjoint Analysis Survey

Scenario attribute	Attribute levels
Reduction in chance of dying from condition because of ED treatment	
Myocardial infarction scenario	Chance of dying decreases from 40% down to 20% Chance of dying decreases from 40% down to 15% Chance of dying decreases from 40% down to 10% Chance of dying decreases from 40% down to 5% Chance of dying decreases from 40% down to 1%
Appendicitis scenario	Chance of dying decreases from 4% down to 2% Chance of dying decreases from 4% down to 1.5% Chance of dying decreases from 4% down to 1% Chance of dying decreases from 4% down to 0.5% Chance of dying decreases from 4% down to 0.1%
Chance of catching COVID-19 in the ED	2% chance of catching COVID-19 1.5% chance of catching COVID-19 1% chance of catching COVID-19 0.5% chance of catching COVID-19 0.1% chance of catching COVID-19
Crowdedness of ED with other patients	Very crowded Somewhat crowded Not at all crowded

Abbreviation: ED, emergency department.

For myocardial infarction and appendicitis scenarios, participants were shown a random set of 10 side-by-side profiles (Figure) drawn from 300 potential sets generated using a balanced overlap design. Moreover, the order in which the 2 scenarios were presented was randomized. Participants were instructed to “choose which [scenario], if any, where you would be more likely to go to the ER for treatment of your [heart attack/appendicitis].” Respondents could have selected neither, thereby choosing to forgo the ED entirely; participants assumed the risk of dying from the myocardial infarction^21^ or appendicitis^22,23^ without treatment but would have a 0% presumed chance of contracting COVID-19 by staying home. Forgoing treatment was presumed to be associated with a 40% risk of death for myocardial infarction^21^ and a 4% risk of death with appendicitis.^22,23^

#### Open-Ended Questions

As considerations other than the attributes presented in the CBC exercises may have influenced respondents’ decisions, we included 2 free-text questions: (1) During the COVID-19 pandemic, what other considerations might you have when deciding whether to go to the ER for treatment that were not listed in the previous sections? (2) In thinking about precautions that ERs take to protect patients from COVID-19, what would you require in order to consider seeking care in the ER?

#### Covariates

The survey collected self-reported sociodemographic information, including age, sex, race/ethnicity, educational attainment, marital status, income, employment status, insurance status, usual source of care, tobacco and alcohol use, household size, number of dependents, and US region. Race/ethnicity was defined using the UCLA Center for Health Policy Research classification of 5 mutually exclusive categories^24^ and was collected given that there may have been differences in decision-making among racial/ethnic groups. Participants were also asked about their self-reported health status,^25^ comorbidities,^26^ religious affiliation, political party affiliation, and news source. We also collected COVID-19–related data, including self-perceived chance of catching COVID-19 and chance of dying if COVID-19 contracted, related anxiety (using the Coronavirus Anxiety Scale^27^), beliefs on wearing face masks in public, whether they knew someone who tested positive for COVID-19, and whether anyone in their household was at increased risk for COVID-19 illness. The Coronavirus Anxiety Scale includes 5 items, each of which is scored on a 0 to 4 Likert scale; accumulated scores range from 0 to 20 (higher = more anxiety related to COVID-19).

### Statistical Analyses

Informed by conjoint analysis sample size precedents and recommendations from the software provider,^28^ we aimed to recruit at least 300 individuals. However, to increase the number of meaningful responses to the open-ended questions, our goal was to recruit closer to 1000 individuals to collect a range of perspectives.

After participants completed CBC exercises, we used hierarchical Bayes regression in conjoint analysis software to estimate individual-level importance scores for each tested attribute^29,30^; attributes with higher scores were more highly valued in decision-making.^30,31^ We also used latent class analysis in this software to identify distinct segments of respondents with similar choice patterns.^32^

We performed statistical analyses on the unweighted data using Stata statistical software version 17.0 (StataCorp). A 2-tailed *P* < .05 was considered statistically significant. Descriptive analyses were used for importance scores and latent classes derived from myocardial infarction and appendicitis exercises. We performed multivariable logistic regression models on prioritizing avoidance of COVID-19 exposure in the ED as determined through latent class analyses for both scenarios. To adjust for confounding, regression models included covariates (see previous section) with a *P* < .10 in bivariate analyses.

Given the large number of collected covariates, we also conducted a sensitivity analysis using a more restrictive *P* < .01 to determine variables for inclusion in the regressions. Results were reported as adjusted odds ratios (aORs) with 95% CIs. For open-ended questions, we used summative content analysis to examine participants’ responses^33^; free texts (word, sentence, and paragraph) were coded and organized into categories and subcategories. Data were analyzed from July 2020 through May 2021.

## Results

### Study Population

Invitations were sent to 1981 individuals by Cint, and 1517 individuals (76.6%) accessed the survey. Among these individuals, we excluded 213 individuals (14.0%) who did not finish the survey, 133 individuals (8.8%) who did not consent to taking the survey, 95 individuals (6.3%) who gave implausible responses (eg, straight-line responses on CBC exercises, bot responses on free-text questions, or impossible answer combinations on multiselect multiple choice questions), 83 individuals (5.5%) who reported a positive COVID-19 test, and 60 individuals (4.0%) aged younger than 18 years.

The analytic set included 933 respondents, for a participation rate of 47.1%; survey completers, compared with 584 noncompleters, were older (mean [SD] age, 40.1 [13.0] years vs 33.4 [16.9] years; *P* < .001) and had a higher proportion of women (491 [52.6%] women vs 222 [48.0%] women; *P* < .001) (eTable 1 in the Supplement). Table 2 presents demographics of the study cohort, and eTable 2 in the Supplement shows comparisons with the US population. Among respondents, 594 individuals (63.7%) self-reported as non-Hispanic White, and 109 individuals (11.7%) self-reported as Latino.

**Table 2.** Demographic Characteristics of Study Population

Characteristic	Participants, No. (%) (N = 933)
Age, y	
18-29	230 (24.7)
30-39	232 (24.9)
40-49	218 (23.4)
50-59	165 (17.7)
≥60	88 (9.4)
Sex	
Men	438 (47.0)
Women	491 (52.6)
Prefer not to say	4 (0.4)
Race/ethnicity	
Non-Hispanic White	594 (63.7)
Non-Hispanic Black	109 (11.7)
Latino	109 (11.7)
Non-Hispanic Asian	71 (7.6)
Other	50 (5.4)
Educational attainment	
High school degree or less	247 (26.5)
Some college education	205 (22.0)
College degree	355 (38.1)
Graduate degree	126 (13.5)
Marital status	
Married or living with a partner	361 (38.7)
Not married	572 (61.3)
Total household income, $	
<50 000	410 (43.9)
50 000-100 000	310 (33.2)
≥100 001	156 (16.7)
Prefer not to say	57 (6.1)
Employment status	
Unemployed, on disability, on leave of absence from work, retired, or a homemaker	365 (39.1)
Employed or student	568 (60.9)
Household size, median (IQR)	3 (2-4)
No. of children or dependents, median (IQR)	0 (0-1)
Current smoking status	
Not at all	686 (73.5)
Some days	71 (7.6)
Every day	176 (18.9)
Typical alcohol use per week, d	
None	495 (53.1)
1-3	322 (34.5)
4-6	78 (8.4)
7	38 (4.1)
Has health insurance	775 (83.1)
Has usual source of care	585 (62.7)
Self-reported health status	
Excellent	194 (20.8)
Very good	312 (33.4)
Good	288 (30.9)
Fair	112 (12.0)
Poor	27 (2.9)
Comorbidities, No.^a^	
0	375 (40.2)
1	198 (21.2)
2	143 (15.3)
≥3	217 (23.3)
Religion	
Buddhist, Christian, Hindu, Jewish, Muslim, or other religion	631 (67.6)
Agnostic or atheist	101 (10.8)
None of the above	160 (17.2)
Prefer not to say	41 (4.4)
Political party affiliation	
Democrat	359 (38.5)
Independent	232 (24.9)
Republican	251 (26.9)
Something else	39 (4.2)
Prefer not to say	52 (5.6)
Main source of news	
National or cable television news	315 (33.8)
Local television news	204 (21.9)
Newspaper	24 (2.6)
Online news websites	147 (15.8)
Social media websites	145 (15.5)
Other source	31 (3.3)
Do not read, watch, or listen to the news	67 (7.2)
US region	
Northeast	179 (19.2)
South	348 (37.3)
Midwest	205 (22.0)
West	201 (21.5)

Abbreviation: IQR, interquartile range.

^a^ Includes heart disease, high blood pressure, lung disease, diabetes, ulcer or stomach disease, kidney disease, liver disease, anemia or other blood disease, cancer, depression, osteoarthritis or degenerative arthritis, back pain, rheumatoid arthritis, or other medical problems.

### COVID-19 Knowledge, Attitudes, and Beliefs

Table 3 presents participants’ responses on their perceived chances of contracting COVID-19 (eg, the largest group, with 293 individuals [31.4%], had perceived odds of ≥10%) and of dying if contracted (eg, the largest group, with 446 individuals [47.8%], had perceived odds of 0%-20%). Individual items from the Coronavirus Anxiety Scale, respondents’ views on wearing face masks, and whether they knew someone diagnosed with COVID-19 are also presented in Table 3.

**Table 3.** COVID-19 Knowledge, Attitudes, Beliefs, and Experiences

Variable	Participants, No. (%) (N = 933)
Perceived chance of contracting COVID-19, %	
≤1	264 (28.3)
2	108 (11.6)
4	111 (11.9)
6	91 (9.8)
8	66 (7.1)
≥10	293 (31.4)
Perceived chance of dying from COVID-19 if caught virus, %	
0-20	446 (47.8)
21-40	184 (19.7)
41-60	131 (14.0)
61-80	121 (13.0)
81-100	51 (5.5)
Coronavirus Anxiety Scale (over previous 2 wk)	
I felt dizzy, lightheaded, or faint when I read or listened to news about the coronavirus	
Not at all	728 (78.0)
Rare, <1-2 d	104 (11.2)
Several days	56 (6.0)
>7 d	27 (2.9)
Nearly every day over the last 2 wk	18 (1.9)
I had trouble falling or staying asleep because I was thinking about the coronavirus	
Not at all	563 (60.3)
Rare, <1-2 d	158 (16.9)
Several days	133 (14.3)
>7 d	51 (5.5)
Nearly every day over the last 2 wk	28 (3.0)
I felt paralyzed or frozen when I thought about or was exposed to information about the coronavirus	
Not at all	729 (78.1)
Rare, <1-2 d	112 (12.0)
Several days	41 (4.4)
>7 d	32 (3.4)
Nearly every day over the last 2 wk	19 (2.0)
I lost interest in eating when I thought about or was exposed to information about the coronavirus	
Not at all	706 (75.7)
Rare, <1-2 d	108 (11.6)
Several days	54 (5.8)
>7 d	42 (4.5)
Nearly every day over the last 2 wk	23 (2.5)
I felt nauseous or had stomach problems when I thought about or was exposed to information about the coronavirus	
Not at all	688 (73.7)
Rare, less than a day or 2	120 (12.9)
Several days	66 (7.1)
>7 d	40 (4.3)
Nearly every day over the last 2 wk	19 (2.0)
Wearing a face mask in public is an important step for protecting other people and slowing the spread of COVID-19	
Strongly disagree	67 (7.2)
Disagree	40 (4.3)
Neither agree nor disagree	118 (12.7)
Agree	257 (27.6)
Strongly agree	451 (48.3)
Know someone who tested positive for COVID-19	256 (27.4)
Relationship to individual(s) who tested positive for COVID-19	
Spouse or partner	16 (6.3)
Child	14 (5.5)
Family member other than spouse or child	68 (26.6)
Friend	115 (44.9)
Colleague	46 (18.0)
Other	33 (12.9)
Someone in household tested positive for COVID-19	221 (23.7)
Someone in household at higher risk for severe COVID-19	270 (28.9)

### Conjoint Analysis to Assess Decision-Making on Emergency Care Seeking During a Pandemic

#### Myocardial Infarction Scenario

For the myocardial infarction scenario, the mean (SD) importance scores were 53.4% (SD 21.8%) for reduction in chance of dying because of ED treatment, 25.0% (17.3%) for crowdedness of ED with other patients, and 21.6% (12.8%) for chance of contracting COVID-19 in the ED. In latent class analysis, 2 distinct groups shared similar choice preferences: 775 individuals (83.1%) prioritized seeking emergency care, and 158 individuals (16.9%) prioritized avoidance of COVID-19 exposure in the ED (ie, chance of contracting COVID-19 in the ED and crowdedness of ED with other patients). Table 4 depicts results from the regression analysis on prioritizing avoidance of COVID-19 exposure. We found higher odds of prioritizing avoidance of COVID-19 exposure among individuals aged 40 to 49 years (aOR vs those aged 18-29 years, 2.42 [95% CI, 1.30-4.52]; *P* = .006), occasional smokers (aOR vs nonsmokers, 2.89 [95% CI, 1.46-5.72]; *P* = .002), and those who did not consume news (aOR vs those with national or cable television news as their main source, 3.44 [95% CI, 1.62-7.31]; *P* = .001). Conversely, we found that respondents were less likely to prioritize avoidance of COVID-19 exposure if they had a usual source of care (aOR, 0.49 [95% CI, 0.32-0.76]; *P* = .001), worse self-reported health status (eg, fair self-reported health status: aOR vs excellent self-reported health status, 0.35 [95% CI, 0.16-0.76]; *P* = .008), higher perceived chance of contracting COVID-19 (eg, perceived chance of ≥10%: aOR vs perceived chance of ≤1%, 0.47 [95% CI, 0.27-0.81]; *P* = .007), or annual household incomes of $100 001 or more (aOR vs annual household income of <$50 000, 0.45 [95% CI, 0.21-0.98]; *P* = .04). The remaining variables largely were not factors associated with decision-making. Results from the regression model using a more restrictive *P* value threshold for determining variable inclusion were similar to those of the primary analysis (eTable 3 in the Supplement).

**Table 4.** Regression Analyses on Prioritizing Avoidance of COVID-19 Exposure

Variable^a^	Prioritized avoidance of COVID-19 exposure in ED			
	Myocardial infarction scenario		Appendicitis scenario	
	aOR (95% CI)	*P* value	aOR (95% CI)	*P* value
Age, y				
18-29	1 [Reference]	NA	1 [Reference]	NA
30-39	1.78 (0.98-3.23)	.06	1.19 (0.73-1.93)	.48
40-49	2.42 (1.30-4.52)	.006	1.05 (0.63-1.76)	.84
50-59	1.69 (0.85-3.36)	.14	0.74 (0.41-1.31)	.30
≥60	0.80 (0.27-2.33)	.68	0.49 (0.22-1.12)	.09
Sex				
Men	1 [Reference]	NA	1 [Reference]	NA
Women	1.33 (0.87-2.02)	.19	0.98 (0.69-1.39)	.90
Race/ethnicity				
Non-Hispanic White	1 [Reference]	NA	1 [Reference]	NA
Non-Hispanic Black	1.46 (0.80-2.66)	.22	1.62 (0.97-2.71)	.06
Latino	0.75 (0.36-1.57)	.45	0.84 (0.47-1.50)	.56
Non-Hispanic Asian	1.41 (0.66-3.02)	.37	1.39 (0.74-2.61)	.31
Other	1.08 (0.46-2.56)	.86	1.29 (0.61-2.72)	.50
Educational attainment				
High school degree or less	1 [Reference]	NA	1 [Reference]	NA
Some college education	0.83 (0.47-1.47)	.52	1.27 (0.80-2.04)	.31
College degree	0.79 (0.46-1.36)	.40	0.81 (0.51-1.29)	.37
Graduate degree	0.92 (0.44-1.94)	.84	0.91 (0.48-1.71)	.76
Marital status				
Married or living with a partner	1 [Reference]	NA	1 [Reference]	NA
Not married	1.04 (0.64-1.68)	.88	0.78 (0.52-1.16)	.22
Total household income, $				
<50 000	1 [Reference]		1 [Reference]	NA
50 000-100 000	0.71 (0.42-1.18)	.19	0.97 (0.64-1.48)	.89
≥100 001	0.45 (0.21-0.98)	.04	0.64 (0.35-1.18)	.15
Prefer not to say	2.40 (1.14-5.07)	.02	2.15 (1.10-4.20)	.03
Employment status				
Unemployed, on disability, on leave of absence from work, retired, or a homemaker	1 [Reference]	NA	1 [Reference]	NA
Employed or student	1.08 (0.69-1.70)	.74	0.99 (0.68-1.46)	.98
Current smoking status				
Not at all	1 [Reference]	NA	1 [Reference]	NA
Some days	2.89 (1.46-5.72)	.002	1.79 (0.97-3.31)	.06
Every day	0.81 (0.46-1.42)	.46	1.10 (0.68-1.76)	.70
Typical alcohol use per week, d				
None	1 [Reference]	NA	1 [Reference]	NA
1-3	0.89 (0.55-1.45)	.65	0.79 (0.53-1.18)	.25
4-6	0.80 (0.36-1.78)	.59	0.78 (0.41-1.48)	.44
7	2.13 (0.86-5.25)	.10	1.04 (0.45-2.44)	.92
Has health insurance	1.38 (0.81-2.37)	.24	0.76 (0.49-1.18)	.22
Has usual source of care	0.49 (0.32-0.76)	.001	0.57 (0.40-0.82)	.003
Self-reported health status				
Excellent	1 [Reference]	NA	1 [Reference]	NA
Very good	0.50 (0.29-0.87)	.02	0.83 (0.52-1.33)	.44
Good	0.52 (0.30-0.90)	.02	0.88 (0.55-1.42)	.61
Fair	0.35 (0.16-0.76)	.008	0.58 (0.30-1.14)	.11
Poor	0.56 (0.18-1.76)	.32	1.34 (0.50-3.62)	.57
Religion				
Buddhist, Christian, Hindu, Jewish, Muslim, or other religion	1 [Reference]	NA	1 [Reference]	NA
Agnostic or atheist	1.36 (0.68-2.71)	.39	0.96 (0.53-1.73)	.88
None of the above	1.63 (0.99-2.68)	.06	1.20 (0.77-1.85)	.42
Prefer not to say	0.47 (0.15-1.47)	.20	0.71 (0.29-1.72)	.44
Political party affiliation				
Democrat	1 [Reference]		1 [Reference]	NA
Independent	0.81 (0.48-1.37)	.43	1.09 (0.71-1.69)	.68
Republican	0.64 (0.36-1.13)	.12	0.70 (0.44-1.12)	.14
Something else	1.21 (0.49-2.95)	.68	0.55 (0.23-1.35)	.19
Prefer not to say	0.82 (0.32-2.14)	.69	1.02 (0.46-2.25)	.96
Main source of news				
National or cable television news	1 [Reference]	NA	1 [Reference]	NA
Local television news	1.30 (0.73-2.29)	.37	1.16 (0.72-1.85)	.54
Newspaper	1.02 (0.24-4.33)	.98	0.32 (0.06-1.56)	.16
Online news websites	0.73 (0.36-1.50)	.40	0.82 (0.47-1.44)	.49
Social media websites	1.15 (0.61-2.14)	.67	1.01 (0.60-1.69)	.98
Other source	2.16 (0.76-6.13)	.15	1.93 (0.78-4.81)	.16
Do not read, watch, or listen to the news	3.44 (1.62-7.31)	.001	1.88 (0.95-3.73)	.07
Perceived chance of contracting COVID-19, %				
≤1	1 [Reference]	NA	1 [Reference]	NA
2	0.46 (0.23-0.94)	.03	0.54 (0.31-0.96)	.04
4	0.25 (0.11-0.56)	.001	0.45 (0.25-0.82)	.009
6	0.57 (0.27-1.21)	.14	0.61 (0.32-1.15)	.13
8	0.43 (0.17-1.09)	.08	0.38 (0.17-0.85)	.02
≥10	0.47 (0.27-0.81)	.007	0.56 (0.35-0.88)	.01
Perceived chance of dying from COVID-19 if caught virus (0%-100%)	1.008 (0.999-1.017)	.08	1.001 (0.994-1.008)	.78
Coronavirus Anxiety Scale score^b^	0.99 (0.94-1.05)	.76	0.98 (0.93-1.03)	.45
Wearing a face mask in public is an important step for protecting other people and slowing the spread of COVID-19				
Strongly disagree	1 [Reference]	NA	1 [Reference]	NA
Disagree	0.73 (0.21-2.48)	.61	0.57 (0.19-1.69)	.31
Neither agree nor disagree	1.71 (0.76-3.85)	.19	1.96 (0.95-4.05)	.07
Agree	0.61 (0.28-1.34)	.22	0.73 (0.37-1.44)	.36
Strongly agree	0.58 (0.28-1.22)	.15	0.76 (0.40-1.46)	.41
Someone in household at increased risk for severe COVID-19	1.34 (0.83-2.17)	.24	1.13 (0.76-1.69)	.55

Abbreviations: aOR, adjusted odds ratio; ED, emergency department; NA, not applicable.

^a^ The multivariable logistic regression models included all covariates in the table; these variables were selected for inclusion in the models given that they had *P* < .10 in bivariate analyses.

^b^ Higher scores mean greater anxiety.

#### Appendicitis Scenario

For the appendicitis scenario, the mean (SD) importance scores were 36.6% (16.6%) for reduction in chance of dying because of ED treatment, 33.3% (17.9%) for crowdedness of ED with other patients, and 30.1% (11.0%) for chance of contracting COVID-19 in the ED. We noted 2 discrete groups in latent class analysis: 695 individuals (74.5%) equally prioritized all 3 attributes, and 238 individuals (25.5%) prioritized avoidance of COVID-19 exposure in the ED. In regression analysis, individuals with a usual source of care (aOR, 0.57 [95% CI, 0.40-0.82]; *P* = .003) and a higher perceived chance of contracting COVID-19 (eg, perceived chance of ≥10%: aOR vs perceived chance of ≤1%, 0.56 [95% CI, 0.35-0.88]; *P* = .01) were less likely to prioritize avoidance of COVID-19 exposure in the ED (Table 4). The remaining variables were not associated with respondents’ preferences. Results from the regression model using the more restrictive *P* value threshold for determining variable inclusion were similar to those of the primary analysis (eTable 3 in the Supplement).

### Qualitative Analyses of Free-Response Questions

Overall, 513 and 639 analyzable responses were provided for the open-ended questions on additional considerations for care seeking and ED precautions, respectively. As for other factors that may have been associated with the decision to seek care, severity of symptoms was most commonly cited (181 individuals [35.3%]). Other considerations included safety measures implemented in the ED (76 individuals [14.8%]), ED wait times (56 individuals [10.9%]), financial considerations (47 individuals [9.2%]), proximity and transportation to the ED (31 individuals [6.0%]), local COVID-19 infection rate (25 individuals [4.9%]), alternatives to in-person care (eg, self-treatment or telehealth availability; 24 individuals [4.7%]), and reputation of the ED or hospital (18 individuals [3.5%]).

When asked about necessary precautions that EDs need to implement, participants reported physical separation from patients with COVID-19 in the waiting room and ED. Respondents also noted that hospital staff should wear personal protective equipment and use hand sanitizer frequently. Other respondents mentioned that patients and staff entering the ED should undergo temperature and symptom screening or rapid tests for COVID-19. Additionally, some participants said they expected facilities to be regularly sanitized.

## Discussion

This survey study assessed the trade-offs individuals made when deciding whether to present to the ED during the COVID-19 pandemic for potentially life-threatening issues unrelated to COVID-19. Even when facing a scenario consistent with myocardial infarction, which carried a 40% risk of death without treatment, 16.9% of individuals prioritized avoidance of COVID-19 exposure in the ED over seeking appropriate care. When presented with a scenario consistent with appendicitis, which carried a 4% risk of death without treatment, 25.5% of individuals chose to forgo emergency care.

Our finding that up to one-quarter of individuals indicated they were willing to forego potentially life-saving treatment during the first peak of the pandemic to avoid exposure to COVID-19 is consistent with prior literature demonstrating significant reductions in hospitalizations for many serious conditions during the pandemic.^2,3,4,5,6,7,8,9,10,11,12,13^ Unlike prior studies that quantified decreases in these admissions, our study determined the relative importance of factors associated with individuals’ decisions on whether to seek care for acute symptoms. Moreover, while we did not present scenarios for routine or preventive care, it stands to reason that even more individuals may be willing to forgo nonemergent care. This assumption is supported by data showing that more than three-quarters of adults nationwide delayed routine medical care during the pandemic.^12,34,35^ While the association of forgone care with emergent issues was identified early in the pandemic,^2,3,4,5,6,7,8,9,10,11,12,13^ the outcomes associated with delayed routine and preventive care will inevitably lead to increased morbidity and mortality over time.^36^ For example, the National Cancer Institute conservatively estimated that over the next decade there will be 10 000 excess deaths associated with delayed screening and treatment of colorectal and breast cancers alone.^37^ These findings suggest that during the current pandemic, as well as during future infectious outbreaks, health care systems and public health organizations should emphasize to patients and the community the importance of continuing to seek care for both emergent and routine issues.

With few exceptions, most sociodemographic factors, political leanings, and beliefs regarding COVID-19 were not factors associated with decision-making in our study. Having a usual source of care, however, was associated with higher odds for preferring to seek appropriate emergency care in both scenarios. The increased inclination to seek treatment among individuals with a usual source of care^38,39^ may be associated with improved access to medical care,^40^ experience in navigating health care systems,^41^ and established relationships with medical providers.^40,42,43,44,45^ These individuals may also have received correspondences from their providers alerting them about the continued importance of seeking care for emergency situations despite the pandemic.^46,47,48,49^ Notably, given that members of racial/ethnic minority groups and those from lower socioeconomic strata are less likely to have a usual source of care,^50,51^ the pandemic is likely exacerbating existing disparities in care unrelated to COVID-19 among these groups.

We also observed that individuals with a higher perceived chance of contracting COVID-19 were more likely to seek appropriate care. While this may appear counterintuitive prima facie, this association may be a surrogate for health-related anxiety; previous research has indicated a positive association between anxiety and health care use.^52,53,54^ Higher perceived risk of contracting COVID-19 may also be associated with unmeasured hypervigilance, thereby leading these individuals to seek treatment when experiencing acute and potentially dangerous symptoms. Further research assessing the association between hypervigilance and care-seeking behaviors for emergent conditions during pandemics is needed.

Our qualitative analyses suggest specific measures that health care facilities and public health organizations can implement to encourage appropriate care seeking and decrease morbidity and mortality associated with delaying care for emergent issues. These results suggest that communications regarding safety precautions that EDs and hospitals are implementing is important, given that many participants indicated this was a major factor in their decisions to seek care. These campaigns should also stress the importance of seeking appropriate care, highlight symptoms that require immediate evaluation, and reinforce the low likelihood of contracting COVID-19 in the ED.^16,46,47,48,49^ Beyond media campaigns, investigators should further examine pathogen-avoidance psychology^14,15^; improved understanding of this maladaptive cognition may inform timely development of novel, effective interventions that encourage appropriate health care seeking during potential future flare-ups of COVID-19, other community-based infectious outbreaks, or any scenario that could promote maladaptive pathogen-avoidance behaviors.

Our study has several strengths. We recruited a sample of nearly 1000 US individuals nationwide and examined the trade-offs they were willing to make when considering presenting for emergency care during a pandemic. We also employed a mixed-methods approach (ie, conjoint analysis, qualitative assessments, and stand-alone multiple-choice questions) to assess the most important factors associated with decision-making.

### Limitations

There were several limitations to our survey study. First, we recruited only US participants, so our findings may not be generalizable to other countries. Second, while the demographics of our study cohort mirrored those of the US population, we had fewer respondents aged 60 years or older than expected, which was likely associated with our use of an online survey. Our results may therefore not generalize to older individuals or to those who lack basic computing skills. Third, we limited the conjoint analysis to 3 attributes, but individuals may have many other considerations when deciding whether to present to the ED. For example, in qualitative analyses, some respondents noted that the local COVID-19 infection rate and reputation of the nearby ED or hospital would be important factors in their decision-making. However, because conjoint surveys can become unwieldy with too many attributes, we opted to focus on 3 core attributes with direct relevance to decision-making in the context of COVID-19. Fourth, despite the design of the conjoint analysis and limitation to 10 vignettes per scenario, the serial decision-making may have been challenging for some participants. We attempted to minimize this issue by consistently presenting probabilities as percentages. Fifth, our survey was conducted in June 2020, during the initial US peak of COVID-19. It is likely that attitudes toward the severity of the pandemic and its association with emergency care seeking behaviors have evolved over time. Nonetheless, the results of a national survey^55^ in December 2020, nearly a year after the first reported cases of COVID-19 in the US, suggested that COVID-19 concerns were still associated with care-seeking behavior; 57% of adults remained hesitant to present to the ED for emergencies. Sixth, respondents were asked to assess hypothetical situations and report how they believe they would act when experiencing real symptoms; their actual decision-making processes may differ. However, a meta-analysis^56^ found that discrete choice experiments are 88% sensitive at predicting health-related choices that individuals make in reality.

## Conclusions

When respondents were faced with hypothetical, serious symptoms, up to 1 in 4 had preferences consistent with forgoing potentially life-saving ED care to avoid COVID-19 exposure. This finding suggests that during the current COVID-19 pandemic and future infectious outbreaks, health care systems and public health organizations should develop communication strategies for patients and the community at large that describe the institutions’ safety measures to reassure and encourage timely health care for critical and routine needs. Moreover, additional research examining pathogen-avoidance psychology is needed to inform development of novel, effective strategies that support appropriate care seeking not only for current and potential future outbreaks of COVID-19, but also for any scenario that could promote maladaptive pathogen-avoidance behaviors.

## Notes

Supplement. **eAppendix 1.** Survey Instrument **eAppendix 2.** Myocardial Infarction Scenario Descriptions **eAppendix 3.** Appendicitis Scenario Descriptions **eTable 1.** Demographic Comparisons Between 1517 Survey Noncompleters and Completers **eTable 2.** Demographics of Study Cohort vs US Population **eTable 3.** Regression Analyses on Prioritizing Avoidance of COVID-19 Exposure With More Restrictive P Value Threshold
