# Supplementary material for: Public Perspectives on Decisions About Emergency Care Seeking for Care Unrelated to COVID-19 During the COVID-19 Pandemic
Source: JAMA Netw Open. 2021 Aug 19;4(8):e2120940. doi: 10.1001/jamanetworkopen.2021.20940 (PMC8377572; doi:10.1001/jamanetworkopen.2021.20940)
Supplement: Supplement. — eAppendix 1. Survey Instrument eAppendix 2. Myocardial Infarction Scenario Descriptions eAppendix 3. Appendicitis Scenario Descriptions eTable 1. Demographic Comparisons Between 1517 Survey Noncompleters and Completers eTable 2. Demographics of Study Cohort vs US Population eTable 3. Regression Analyses on Prioritizing Avoidance of COVID-19 Exposure With More Restrictive P Value Threshold [file jamanetwopen-e2120940-s001.pdf]

Supplemental Online Content

Gale R, Eberlein S, Fuller G, Khalil C, Almario CV, Spiegel BMR. Public perspectives on decisions about emergency care seeking for care unrelated to COVID-19 during the COVID-19 pandemic. *JAMA Netw Open*. 2021;4(8):e2120940. doi:10.1001/jamanetworkopen.2021.20940

eAppendix 1. Survey Instrument
eAppendix 2. Myocardial Infarction Scenario Descriptions
eAppendix 3. Appendicitis Scenario Descriptions
eTable 1. Demographic Comparisons Between 1517 Survey Noncompleters and Completers
eTable 2. Demographics of Study Cohort vs US Population
eTable 3. Regression Analyses on Prioritizing Avoidance of COVID-19 Exposure With More Restrictive P Value Threshold

This supplemental material has been provided by the authors to give readers additional information about their work.

© 2021 Gale R et al. *JAMA Network Open.*

eAppendix 1. Survey Instrument

Start

Examining How People Decide to Go to the Emergency Room During the COVID-19 Pandemic

Welcome and thank you for choosing to participate in our survey.

Our goal is to learn how people make decisions about whether to go to the emergency room (ER) for treatment during the COVID-19 pandemic. On the following few pages, we will first ask some questions to see if you are eligible for the study. If you are eligble, the survey should take about 10-15 minutes.

Let's get started!

→

0% 100%

covidexperience

Which of the following best describes your experience with COVID-19?

covidexperience=1 I received a test for COVID-19 that came back positive

covidexperience=2 I received a test for COVID-19 that came back negative

covidexperience=3 I was not tested for COVID-19 but suspect I had it

covidexperience=4 I was not tested for COVID-19 and do not think I had it

© 2021 Gale R et al. *JAMA Network Open.*

0% 100%

Age

Please enter your age in years:

→

© 2021 Gale R et al. *JAMA Network Open.*

0% 100%

Sex

What is your sex?

Sex=1 Male

Sex=2 Female

Sex=3 Prefer not to say

© 2021 Gale R et al. *JAMA Network Open*.

0% 100%

eligbility

Congratulations, you qualified for our survey!

To continue with the survey, you will first need to review the study information sheet on the next page.

Click the arrow button to continue.

→

© 2021 Gale R et al. *JAMA Network Open.*

0% 100%

Consent

Online Consent/Authorization

Christopher Almario, MD, MSHPM, Principal Investigator
310-423-4462
christopher.almario@csmc.edu

Study Title: Assessing Patient Decision Making When Deciding Whether to Present to the Emergency Department During the Coronavirus Pandemic

You are being invited to complete a survey that is part of a research study conducted by Dr. Christopher Almario at Cedars-Sinai Medical Center (CSMC).

This research study is unsponsored.

The goal of this study is to assess how individuals make decisions on whether to go to the emergency room (ER) for treatment of their acute symptoms during the COVID-19 pandemic.

About 1,000 people will take part in this study at CSMC.

Procedures

You will be asked to complete a questionnaire. We will ask you questions to evaluate what matters most to you when considering to go to the ER for management of your symptoms. We think it should take about 10-15 minutes to complete the questionnaire. We will present you with several hypothetical scenarios and you will choose which one, if any, where you would be more likely to go to the ER for treatment. If you feel uncomfortable or embarrassed answering any question, you may skip it. The questionnaire will not contain any information that could be used to identify you.

Participant Requirements

Participation in this study is limited to individuals age 18 and older with access to the internet and have not been diagnosed with COVID-19.

Risks

There might be some possible risks or discomforts associated with taking part in this study. It is possible that some of the items in the questionnaire may make you feel uncomfortable or embarrassed. You are not required to respond to any item that you do not wish to answer.

Benefits

While no benefit is ever guaranteed, we hope the information learned from this research study will benefit other individuals in the future by helping us to learn what factors patients consider important when seeking emergency care during a pandemic.

© 2021 Gale R et al. *JAMA Network Open.*

Collection of Health Information

If you decide to take part in this study, you are giving the research team permission to collect your health information through this survey and store this information for use in this study. We will not collect your name and all identifying information will be removed from research data so that no one can tell which information is yours. Your personal identification will **never** be mentioned in any publication or communication of the research data. Private health information to be collected about you in this survey will include:

- Other types of medical information about you such as your age, race/ethnicity, gender, and chronic conditions.

Every reasonable effort will be made to keep your records confidential. Access to your information is limited to that which is minimally essential to conduct the study and is given only to the investigators and authorized research staff.

In addition to the investigators and their research team, the following parties may receive information about you from the study:

- Medical and other health care professional students who are assisting with tasks for the research study.

By agreeing to participate in this study, you authorize the use and sharing of your health information, as explained in this document, through December 31, 2022.

Confidentiality

CSMC respects your private information and adheres to the Health Insurance Portability and Accountability Act (HIPAA) and other federal and state laws that protect your privacy.

Information collected about you during the course of this research may be subject to inspection by CSMC departments and committees responsible for research oversight, accrediting agencies, government and regulatory groups (e.g. Food and Drug Administration (FDA), Office for Human Research Protections (OHRP), etc.), safety monitors, and the Study Sponsor.

Once you have completed this survey, your information will be transmitted over the internet to CSMC. Although every reasonable effort has been taken to ensure that your information is encrypted, confidentiality during the actual internet communication procedure cannot be guaranteed.

The data and information gathered during this study may be used by the investigator and published and/or disclosed outside of CSMC in publications or other dissemination of the research data. In these cases, however, your personal identifiers will not be mentioned in any publication or dissemination of the research data and/or results by the investigator.

Right to Ask Questions & Contact Information

If you have any questions about this consent form or the study, you should feel free to ask them by contacting the Principal Investigator by mail, phone or e-mail as noted in the contact information listed above. If you use e-mail, you should be aware that confidentiality during the transmission process cannot be guaranteed. Therefore, we do not suggest using email to relay any private or

© 2021 Gale R et al. *JAMA Network Open.*

sensitive information.

If you have questions regarding your rights, concerns, or complaints about taking part in this study, please contact:

Cedars-Sinai Institutional Review Board (IRB)
Phone: (310) 423-3783
Email: ResearchConcerns@cshs.org

The Cedars-Sinai IRB has been established to review, approve, and monitor all human research at CSMC with the purpose of minimizing risks and protecting the rights and welfare of research participants.

Voluntary Participation

Your participation in this research is voluntary. You may discontinue participation at any time.

If you have any questions or concerns about the information in this consent form or about this study, you should not agree to participate until all of your questions have been answered. Please contact the following individual before proceeding:

Christopher V. Almario, MD, MSHPM
Principal Investigator
310-423-4462

→

© 2021 Gale R et al. *JAMA Network Open.*

0% 100%

Consent1

I have read and understand the information on the previous page and am ready to complete this form without further discussion.

Consent1=1 Yes

Consent1=2 No

Consent2

I have read and understand that every reasonable effort will be made to keep my information confidential; however absolute confidentiality cannot be guaranteed.

Consent2=1 Yes

Consent2=2 No

Consent3

I agree to participate in this research and continue with the survey.

Consent3=1 Yes

Consent3=2 No

0% 100%

PerceivedCatchingC19

In general, what is your perceived chance of catching COVID-19?

PerceivedCatchingC19=1
1% or less

PerceivedCatchingC19=2
2%

PerceivedCatchingC19=3
4%

PerceivedCatchingC19=4
6%

PerceivedCatchingC19=5
8%

PerceivedCatchingC19=6
10% or more

© 2021 Gale R et al. *JAMA Network Open.*

0% 100%

PerceivedC19mortality

If you were to catch COVID-19, what is your perceived chance of dying from it?

0% 20% 40% 60% 80% 100%

© 2021 Gale R et al. *JAMA Network Open.*

0% 100%

covidanxiety

How often have you experienced the following activities over the last 2 weeks?

	Not at all	Rare, less than a day or two	Several days	More than 7 days	Nearly every day over the last 2 weeks
I felt dizzy, lightheaded, or faint, when I read or listened to news about the coronavirus.	covidanxiety_r1=1 ◯	covidanxiety_r1=2 ◯	covidanxiety_r1=3 ◯	covidanxiety_r1=4 ◯	covidanxiety_r1=5 ◯
I had trouble falling or staying asleep because I was thinking about the coronavirus.	covidanxiety_r2=1 ◯	covidanxiety_r2=2 ◯	covidanxiety_r2=3 ◯	covidanxiety_r2=4 ◯	covidanxiety_r2=5 ◯
I felt paralyzed or frozen when I thought about or was exposed to information about the coronavirus.	covidanxiety_r3=1 ◯	covidanxiety_r3=2 ◯	covidanxiety_r3=3 ◯	covidanxiety_r3=4 ◯	covidanxiety_r3=5 ◯
I lost interest in eating when I thought about or was exposed to information about the coronavirus.	covidanxiety_r4=1 ◯	covidanxiety_r4=2 ◯	covidanxiety_r4=3 ◯	covidanxiety_r4=4 ◯	covidanxiety_r4=5 ◯
I felt nauseous or had stomach problems when I thought about or was exposed to information about the	covidanxiety_r5=1 ◯	covidanxiety_r5=2 ◯	covidanxiety_r5=3 ◯	covidanxiety_r5=4 ◯	covidanxiety_r5=5 ◯

© 2021 Gale R et al. *JAMA Network Open.*

coronavirus.

© 2021 Gale R et al. *JAMA Network Open.*

0% 100%

conjointtransition

Examining How People Decide to Go to the Emergency Room During the COVID-19 Pandemic

Next, we want to learn how you think about the risks and benefits of visiting the ER for treatment during the COVID-19 pandemic.

On the next page you will see a description of a situation where you must decide whether or not to visit the ER.

→

© 2021 Gale R et al. *JAMA Network Open.*

0% 100%

conjointattributesHA1

Experiencing Symptoms Likely Related to a Heart Attack

For the next few pages, imagine that you are experiencing chest pain that came on suddenly. The pain is likely related to a **heart attack and the risk of dying from it without treatment is 40%**. Receiving treatment in the ER will reduce your risk of dying from a heart attack. However, going to the ER may potentially expose you to others who have COVID-19.

Below are three considerations that may affect your decision about whether or not to visit the ER. Please read the information carefully as it will help as you go through the next exercise.

Reduction in Chance of Dying from Heart Attack because of ER Treatment
This is how much your chance of dying from a heart attack goes down if you receive treatment in the ER.

Chance of Catching COVID-19 in the ER
This is the chance of catching COVID-19 in the ER from other patients, doctors, nurses, or other members of the medical team.

Crowdedness of ER with Other Patients
This represents how crowded the ER is with other patients who are seeking care.

→

© 2021 Gale R et al. *JAMA Network Open.*

0% 100%

CBCChest_Random1

INSTRUCTIONS: Imagine that you are experiencing chest pain that came on suddenly. The pain is likely related to a heart attack and the risk of dying from it without treatment is 40%.

Read the two scenarios below and choose which one, if any, where you would be more likely to go to the ER for treatment of your heart attack?

(1 of 10)

	**Scenario A**	**Scenario B**
Reduction in chance of dying from heart attack because of ER treatment	Chance of dying decreases from 40% down to 15%	Chance of dying decreases from 40% down to 5%
Chance of catching COVID-19 in the ER	1.5% chance of catching COVID-19	0.5% chance of catching COVID-19
Crowdedness of ER with other patients	Very crowded	Somewhat crowded
	CBCChest_Random1 Select	CBCChest_Random1 Select

Neither

I would not go to the ER in either scenario and would rather stay at home.

My chance of dying from heart attack is 40%,
and my chance of catching COVID-19 is 0%.

CBCChest_Random1

Select

→

© 2021 Gale R et al. *JAMA Network Open.*

0% 100%

CBCChest_Random2

INSTRUCTIONS: Imagine that you are experiencing chest pain that came on suddenly. The pain is likely related to a heart attack and the risk of dying from it without treatment is 40%.

Read the two scenarios below and choose which one, if any, where you would be more likely to go to the ER for treatment of your heart attack?

(2 of 10)

	**Scenario A**	**Scenario B**
Reduction in chance of dying from heart attack because of ER treatment	Chance of dying decreases from 40% down to 15%	Chance of dying decreases from 40% down to 5%
Chance of catching COVID-19 in the ER	1% chance of catching COVID-19	2% chance of catching COVID-19
Crowdedness of ER with other patients	Somewhat crowded	Not at all crowded
	CBCChest_Random2 Select	CBCChest_Random2 Select

Neither

I would not go to the ER in either scenario and would rather stay at home.

My chance of dying from heart attack is 40%,
and my chance of catching COVID-19 is 0%.

CBCChest_Random2

Select

→

© 2021 Gale R et al. *JAMA Network Open.*

0% 100%

CBCChest_Random3

INSTRUCTIONS: Imagine that you are experiencing chest pain that came on suddenly. The pain is likely related to a heart attack and the risk of dying from it without treatment is 40%.

Read the two scenarios below and choose which one, if any, where you would be more likely to go to the ER for treatment of your heart attack?

(3 of 10)

	Scenario A	Scenario B
Reduction in chance of dying from heart attack because of ER treatment	Chance of dying decreases from 40% down to 15%	Chance of dying decreases from 40% down to 1%
Chance of catching COVID-19 in the ER	0.1% chance of catching COVID-19	0.1% chance of catching COVID-19
Crowdedness of ER with other patients	Very crowded	Very crowded
	CBCChest_Random3 Select	CBCChest_Random3 Select

Neither

I would not go to the ER in either scenario and would rather stay at home.

My chance of dying from heart attack is 40%,
and my chance of catching COVID-19 is 0%.

CBCChest_Random3

Select

→

© 2021 Gale R et al. *JAMA Network Open.*

0% 100%

CBCChest_Random4

INSTRUCTIONS: Imagine that you are experiencing chest pain that came on suddenly. The pain is likely related to a heart attack and the risk of dying from it without treatment is 40%.

Read the two scenarios below and choose which one, if any, where you would be more likely to go to the ER for treatment of your heart attack?

(4 of 10)

	**Scenario A**	**Scenario B**
Reduction in chance of dying from heart attack because of ER treatment	Chance of dying decreases from 40% down to 20%	Chance of dying decreases from 40% down to 5%
Chance of catching COVID-19 in the ER	2% chance of catching COVID-19	1.5% chance of catching COVID-19
Crowdedness of ER with other patients	Very crowded	Very crowded
	CBCChest_Random4 Select	CBCChest_Random4 Select

Neither

I would not go to the ER in either scenario and would rather stay at home.

My chance of dying from heart attack is 40%,
and my chance of catching COVID-19 is 0%.

CBCChest_Random4
Select

→

© 2021 Gale R et al. *JAMA Network Open.*

0% 100%

CBCChest_Random5

INSTRUCTIONS: Imagine that you are experiencing chest pain that came on suddenly. The pain is likely related to a heart attack and the risk of dying from it without treatment is 40%.

Read the two scenarios below and choose which one, if any, where you would be more likely to go to the ER for treatment of your heart attack?

(5 of 10)

	Scenario A	Scenario B
Reduction in chance of dying from heart attack because of ER treatment	Chance of dying decreases from 40% down to 1%	Chance of dying decreases from 40% down to 1%
Chance of catching COVID-19 in the ER	1.5% chance of catching COVID-19	2% chance of catching COVID-19
Crowdedness of ER with other patients	Somewhat crowded	Somewhat crowded
	CBCChest_Random5 Select	CBCChest_Random5 Select

Neither

I would not go to the ER in either scenario and would rather stay at home.

My chance of dying from heart attack is 40%,
and my chance of catching COVID-19 is 0%.

CBCChest_Random5

Select

→

© 2021 Gale R et al. *JAMA Network Open.*

0% 100%

CBCChest_Random6

INSTRUCTIONS: Imagine that you are experiencing chest pain that came on suddenly. The pain is likely related to a heart attack and the risk of dying from it without treatment is 40%.

Read the two scenarios below and choose which one, if any, where you would be more likely to go to the ER for treatment of your heart attack?

(6 of 10)

	Scenario A	Scenario B
Reduction in chance of dying from heart attack because of ER treatment	Chance of dying decreases from 40% down to 10%	Chance of dying decreases from 40% down to 1%
Chance of catching COVID-19 in the ER	0.5% chance of catching COVID-19	0.1% chance of catching COVID-19
Crowdedness of ER with other patients	Somewhat crowded	Very crowded
	CBCChest_Random6 Select	CBCChest_Random6 Select

Neither

I would not go to the ER in either scenario and would rather stay at home.

My chance of dying from heart attack is 40%,
and my chance of catching COVID-19 is 0%.

CBCChest_Random6

Select

→

© 2021 Gale R et al. *JAMA Network Open.*

0% 100%

CBCChest_Random7

INSTRUCTIONS: Imagine that you are experiencing chest pain that came on suddenly. The pain is likely related to a heart attack and the risk of dying from it without treatment is 40%.

Read the two scenarios below and choose which one, if any, where you would be more likely to go to the ER for treatment of your heart attack?

(7 of 10)

	Scenario A	Scenario B
Reduction in chance of dying from heart attack because of ER treatment	Chance of dying decreases from 40% down to 20%	Chance of dying decreases from 40% down to 5%
Chance of catching COVID-19 in the ER	1% chance of catching COVID-19	0.1% chance of catching COVID-19
Crowdedness of ER with other patients	Somewhat crowded	Very crowded
	CBCChest_Random7 Select	CBCChest_Random7 Select

Neither

I would not go to the ER in either scenario and would rather stay at home.

My chance of dying from heart attack is 40%,
and my chance of catching COVID-19 is 0%.

CBCChest_Random7

Select

© 2021 Gale R et al. *JAMA Network Open.*

0% 100%

CBCChest_Random8

INSTRUCTIONS: Imagine that you are experiencing chest pain that came on suddenly. The pain is likely related to a heart attack and the risk of dying from it without treatment is 40%.

Read the two scenarios below and choose which one, if any, where you would be more likely to go to the ER for treatment of your heart attack?

(8 of 10)

	**Scenario A**	**Scenario B**
Reduction in chance of dying from heart attack because of ER treatment	Chance of dying decreases from 40% down to 15%	Chance of dying decreases from 40% down to 20%
Chance of catching COVID-19 in the ER	1% chance of catching COVID-19	2% chance of catching COVID-19
Crowdedness of ER with other patients	Very crowded	Not at all crowded
	CBCChest_Random8 Select	CBCChest_Random8 Select

Neither

I would not go to the ER in either scenario and would rather stay at home.

My chance of dying from heart attack is 40%,
and my chance of catching COVID-19 is 0%.

CBCChest_Random8

Select

→

© 2021 Gale R et al. *JAMA Network Open.*

0% 100%

CBCChest_Random9

INSTRUCTIONS: Imagine that you are experiencing chest pain that came on suddenly. The pain is likely related to a heart attack and the risk of dying from it without treatment is 40%.

Read the two scenarios below and choose which one, if any, where you would be more likely to go to the ER for treatment of your heart attack?

(9 of 10)

	Scenario A	Scenario B
Reduction in chance of dying from heart attack because of ER treatment	Chance of dying decreases from 40% down to 5%	Chance of dying decreases from 40% down to 10%
Chance of catching COVID-19 in the ER	1% chance of catching COVID-19	1% chance of catching COVID-19
Crowdedness of ER with other patients	Not at all crowded	Somewhat crowded
	CBCChest_Random9 Select	CBCChest_Random9 Select

Neither

I would not go to the ER in either scenario and would rather stay at home.

My chance of dying from heart attack is 40%,
and my chance of catching COVID-19 is 0%.

CBCChest_Random9

Select

→

© 2021 Gale R et al. *JAMA Network Open.*

0% 100%

CBCChest_Random10

INSTRUCTIONS: Imagine that you are experiencing chest pain that came on suddenly. The pain is likely related to a heart attack and the risk of dying from it without treatment is 40%.

Read the two scenarios below and choose which one, if any, where you would be more likely to go to the ER for treatment of your heart attack?

(10 of 10)

	**Scenario A**	**Scenario B**
Reduction in chance of dying from heart attack because of ER treatment	Chance of dying decreases from 40% down to 15%	Chance of dying decreases from 40% down to 10%
Chance of catching COVID-19 in the ER	2% chance of catching COVID-19	2% chance of catching COVID-19
Crowdedness of ER with other patients	Very crowded	Somewhat crowded
	CBCChest_Random10 Select	CBCChest_Random10 Select

Neither

I would not go to the ER in either scenario and would rather stay at home.

My chance of dying from heart attack is 40%,
and my chance of catching COVID-19 is 0%.

CBCChest_Random10

Select

→

© 2021 Gale R et al. *JAMA Network Open.*

0% 100%

conjointattributesAPPY

Experiencing Symptoms Likely Related to Appendicitis

For the next few pages, imagine that you are experiencing belly pain that came on suddenly. The pain is likely related to **appendicitis (ie, inflammation of the appendix) and the risk of dying from it without treatment is 4%.** Receiving treatment in the ER will reduce your risk of dying from appendicitis. However, going to the ER may potentially expose you to others who have COVID-19.

Below are three considerations that may affect your decision about whether or not to visit the ER. Please read the information carefully as it will help as you go through the next exercise.

Reduction in Chance of Dying from Appendicitis because of ER Treatment
This is how much your chance of dying from appendicitis goes down if you receive treatment in the ER.

Chance of Catching COVID-19 in the ER
This is the chance of catching COVID-19 in the ER from other patients, doctors, nurses, or other members of the medical team.

Crowdedness of ER with Other Patients
This represents how crowded the ER is with other patients who are seeking care.

→

© 2021 Gale R et al. *JAMA Network Open.*

0% 100%

CBCBelly_Random1

INSTRUCTIONS: Imagine that you are experiencing belly pain that came on suddenly. The pain is likely related to appendicitis (ie, inflammation of the appendix), and the risk of dying from it without treatment is 4%.

Read the two scenarios below and choose which one, if any, where you would be more likely to go to the ER for treatment of your appendicitis?

(1 of 10)

	Scenario A	Scenario B
Reduction in chance of dying from appendicitis because of ER treatment	Chance of dying decreases from 4% down to 1.5%	Chance of dying decreases from 4% down to 0.5%
Chance of catching COVID-19 in the ER	1.5% chance of catching COVID-19	0.5% chance of catching COVID-19
Crowdedness of ER with other patients	Very crowded	Somewhat crowded
	CBCBelly_Random1 Select	CBCBelly_Random1 Select

Neither

I would not go to the ER in either scenario and would rather stay at home.

My chance of dying from appendicitis is 4%,
and my chance of catching COVID-19 is 0%.

CBCBelly_Random1

Select

→

© 2021 Gale R et al. *JAMA Network Open.*

0% 100%

CBCBelly_Random2

INSTRUCTIONS: Imagine that you are experiencing belly pain that came on suddenly. The pain is likely related to appendicitis (ie, inflammation of the appendix), and the risk of dying from it without treatment is 4%.

Read the two scenarios below and choose which one, if any, where you would be more likely to go to the ER for treatment of your appendicitis?

(2 of 10)

	Scenario A	Scenario B
Reduction in chance of dying from appendicitis because of ER treatment	Chance of dying decreases from 4% down to 1.5%	Chance of dying decreases from 4% down to 0.5%
Chance of catching COVID-19 in the ER	1% chance of catching COVID-19	2% chance of catching COVID-19
Crowdedness of ER with other patients	Somewhat crowded	Not at all crowded
	CBCBelly_Random2 Select	CBCBelly_Random2 Select

Neither

I would not go to the ER in either scenario and would rather stay at home.

My chance of dying from appendicitis is 4%,
and my chance of catching COVID-19 is 0%.

CBCBelly_Random2

Select

→

© 2021 Gale R et al. *JAMA Network Open.*

0% 100%

CBCBelly_Random3

INSTRUCTIONS: Imagine that you are experiencing belly pain that came on suddenly. The pain is likely related to appendicitis (ie, inflammation of the appendix), and the risk of dying from it without treatment is 4%.

Read the two scenarios below and choose which one, if any, where you would be more likely to go to the ER for treatment of your appendicitis?

(3 of 10)

	Scenario A	Scenario B
Reduction in chance of dying from appendicitis because of ER treatment	Chance of dying decreases from 4% down to 1.5%	Chance of dying decreases from 4% down to 0.1%
Chance of catching COVID-19 in the ER	0.1% chance of catching COVID-19	0.1% chance of catching COVID-19
Crowdedness of ER with other patients	Very crowded	Very crowded
	CBCBelly_Random3 Select	CBCBelly_Random3 Select

Neither

I would not go to the ER in either scenario and would rather stay at home.

My chance of dying from appendicitis is 4%,
and my chance of catching COVID-19 is 0%.

CBCBelly_Random3

Select

→

© 2021 Gale R et al. *JAMA Network Open.*

0% 100%

CBCBelly_Random4

INSTRUCTIONS: Imagine that you are experiencing belly pain that came on suddenly. The pain is likely related to appendicitis (ie, inflammation of the appendix), and the risk of dying from it without treatment is 4%.

Read the two scenarios below and choose which one, if any, where you would be more likely to go to the ER for treatment of your appendicitis?

(4 of 10)

	**Scenario A**	**Scenario B**
Reduction in chance of dying from appendicitis because of ER treatment	Chance of dying decreases from 4% down to 2%	Chance of dying decreases from 4% down to 0.5%
Chance of catching COVID-19 in the ER	2% chance of catching COVID-19	1.5% chance of catching COVID-19
Crowdedness of ER with other patients	Very crowded	Very crowded
	CBCBelly_Random4 Select	CBCBelly_Random4 Select

Neither

I would not go to the ER in either scenario and would rather stay at home.

My chance of dying from appendicitis is 4%,
and my chance of catching COVID-19 is 0%.

CBCBelly_Random4

Select

→

© 2021 Gale R et al. *JAMA Network Open.*

0% 100%

CBCBelly_Random5

INSTRUCTIONS: Imagine that you are experiencing belly pain that came on suddenly. The pain is likely related to appendicitis (ie, inflammation of the appendix), and the risk of dying from it without treatment is 4%.

Read the two scenarios below and choose which one, if any, where you would be more likely to go to the ER for treatment of your appendicitis?

(5 of 10)

	**Scenario A**	**Scenario B**
Reduction in chance of dying from appendicitis because of ER treatment	Chance of dying decreases from 4% down to 0.1%	Chance of dying decreases from 4% down to 0.1%
Chance of catching COVID-19 in the ER	1.5% chance of catching COVID-19	2% chance of catching COVID-19
Crowdedness of ER with other patients	Somewhat crowded	Somewhat crowded
	CBCBelly_Random5 Select	CBCBelly_Random5 Select

Neither

I would not go to the ER in either scenario and would rather stay at home.

My chance of dying from appendicitis is 4%,
and my chance of catching COVID-19 is 0%.

CBCBelly_Random5

Select

→

© 2021 Gale R et al. *JAMA Network Open.*

0% 100%

CBCBelly_Random6

INSTRUCTIONS: Imagine that you are experiencing belly pain that came on suddenly. The pain is likely related to appendicitis (ie, inflammation of the appendix), and the risk of dying from it without treatment is 4%.

Read the two scenarios below and choose which one, if any, where you would be more likely to go to the ER for treatment of your appendicitis?

(6 of 10)

	Scenario A	Scenario B
Reduction in chance of dying from appendicitis because of ER treatment	Chance of dying decreases from 4% down to 1%	Chance of dying decreases from 4% down to 0.1%
Chance of catching COVID-19 in the ER	0.5% chance of catching COVID-19	0.1% chance of catching COVID-19
Crowdedness of ER with other patients	Somewhat crowded	Very crowded
	CBCBelly_Random6 Select	CBCBelly_Random6 Select

Neither

I would not go to the ER in either scenario and would rather stay at home.

My chance of dying from appendicitis is 4%,
and my chance of catching COVID-19 is 0%.

CBCBelly_Random6

Select

→

© 2021 Gale R et al. *JAMA Network Open*.

0% 100%

CBCBelly_Random7

INSTRUCTIONS: Imagine that you are experiencing belly pain that came on suddenly. The pain is likely related to appendicitis (ie, inflammation of the appendix), and the risk of dying from it without treatment is 4%.

Read the two scenarios below and choose which one, if any, where you would be more likely to go to the ER for treatment of your appendicitis?

(7 of 10)

	**Scenario A**	**Scenario B**
Reduction in chance of dying from appendicitis because of ER treatment	Chance of dying decreases from 4% down to 2%	Chance of dying decreases from 4% down to 0.5%
Chance of catching COVID-19 in the ER	1% chance of catching COVID-19	0.1% chance of catching COVID-19
Crowdedness of ER with other patients	Somewhat crowded	Very crowded
	CBCBelly_Random7 Select	CBCBelly_Random7 Select

Neither

I would not go to the ER in either scenario and would rather stay at home.

My chance of dying from appendicitis is 4%,
and my chance of catching COVID-19 is 0%.

CBCBelly_Random7

Select

→

© 2021 Gale R et al. *JAMA Network Open.*

0% 100%

CBCBelly_Random8

INSTRUCTIONS: Imagine that you are experiencing belly pain that came on suddenly. The pain is likely related to appendicitis (ie, inflammation of the appendix), and the risk of dying from it without treatment is 4%.

Read the two scenarios below and choose which one, if any, where you would be more likely to go to the ER for treatment of your appendicitis?

(8 of 10)

	Scenario A	Scenario B
Reduction in chance of dying from appendicitis because of ER treatment	Chance of dying decreases from 4% down to 1.5%	Chance of dying decreases from 4% down to 2%
Chance of catching COVID-19 in the ER	1% chance of catching COVID-19	2% chance of catching COVID-19
Crowdedness of ER with other patients	Very crowded	Not at all crowded
	CBCBelly_Random8 Select	CBCBelly_Random8 Select

Neither

I would not go to the ER in either scenario and would rather stay at home.

My chance of dying from appendicitis is 4%,
and my chance of catching COVID-19 is 0%.

CBCBelly_Random8
Select

→

© 2021 Gale R et al. *JAMA Network Open*.

0% 100%

CBCBelly_Random9

INSTRUCTIONS: Imagine that you are experiencing belly pain that came on suddenly. The pain is likely related to appendicitis (ie, inflammation of the appendix), and the risk of dying from it without treatment is 4%.

Read the two scenarios below and choose which one, if any, where you would be more likely to go to the ER for treatment of your appendicitis?

(9 of 10)

	**Scenario A**	**Scenario B**
Reduction in chance of dying from appendicitis because of ER treatment	Chance of dying decreases from 4% down to 0.5%	Chance of dying decreases from 4% down to 1%
Chance of catching COVID-19 in the ER	1% chance of catching COVID-19	1% chance of catching COVID-19
Crowdedness of ER with other patients	Not at all crowded	Somewhat crowded
	CBCBelly_Random9 Select	CBCBelly_Random9 Select

Neither

I would not go to the ER in either scenario and would rather stay at home.

My chance of dying from appendicitis is 4%,
and my chance of catching COVID-19 is 0%.

CBCBelly_Random9

Select

→

© 2021 Gale R et al. *JAMA Network Open.*

0% 100%

CBCBelly_Random10

INSTRUCTIONS: Imagine that you are experiencing belly pain that came on suddenly. The pain is likely related to appendicitis (ie, inflammation of the appendix), and the risk of dying from it without treatment is 4%.

Read the two scenarios below and choose which one, if any, where you would be more likely to go to the ER for treatment of your appendicitis?

(10 of 10)

	**Scenario A**	**Scenario B**
Reduction in chance of dying from appendicitis because of ER treatment	Chance of dying decreases from 4% down to 1.5%	Chance of dying decreases from 4% down to 1%
Chance of catching COVID-19 in the ER	2% chance of catching COVID-19	2% chance of catching COVID-19
Crowdedness of ER with other patients	Very crowded	Somewhat crowded
	CBCBelly_Random10 Select	CBCBelly_Random10 Select

Neither

I would not go to the ER in either scenario and would rather stay at home.

My chance of dying from appendicitis is 4%,
and my chance of catching COVID-19 is 0%.

CBCBelly_Random10

Select

→

© 2021 Gale R et al. *JAMA Network Open.*

0% 100%

AnythingMissing

During the COVID-19 pandemic, what other considerations might you have when deciding whether or not to go to the ER for treatment that were not listed in the previous sections?

→

© 2021 Gale R et al. *JAMA Network Open.*

0% 100%

freeresp1

In thinking about precautions that ERs take to protect patients from COVID-19, what would you require in order to consider seeking care in the ER?

→

© 2021 Gale R et al. *JAMA Network Open.*

0% 100%

covidmask

Please indicate how much you agree or disagree with the following statement.

Wearing a face mask in public is an important step for protecting other people and slowing the spread of COVID-19.

- covidmask=1 Strongly disagree
- covidmask=2 Disagree
- covidmask=3 Neither agree or disagree
- covidmask=4 Agree
- covidmask=5 Strongly agree

→

© 2021 Gale R et al. *JAMA Network Open*.

0% 100%

CovidContact1

Do you personally know someone who tested positive with COVID-19?

CovidContact1=1 Yes

CovidContact1=2 No

→

© 2021 Gale R et al. *JAMA Network Open.*

0% 100%

CovidContactHousehold

Did someone in your household test positive with COVID-19?

CovidContactHousehold=1 Yes

CovidContactHousehold=2 No

© 2021 Gale R et al. *JAMA Network Open.*

0% 100%

CovidContact2

Which of the following best describes the person(s) you know who tested positive with COVID-19? (select all that apply)

CovidContact2_1 ☐ Your spouse or partner

CovidContact2_2 ☐ Your child/children

CovidContact2_3 ☐ Your family member(s) other than your spouse or child

CovidContact2_4 ☐ Your friend(s)

CovidContact2_5 ☐ Your colleague(s)

CovidContact2_6 ☐ Other (please specify) CovidContact2_6_other ________

→

© 2021 Gale R et al. *JAMA Network Open*.

0% 100%

demotransition

We are almost done. The final few pages will have questions that focus on your general health and background.

→

0% 100%

Race

How do you describe your race? (select all that apply)

- Race_1 ☐ White
- Race_2 ☐ Black or African-American
- Race_5 ☐ Native Hawaiian or Other Pacific Islander
- Race_3 ☐ Asian
- Race_4 ☐ American Indian or Alaska Native
- Race_6 ☐ Race_6_other Other (please specify) ________
- Race_7 ☐ Prefer not to say

Ethnicity

Are you of Spanish, Hispanic, or Latino origin?

- Ethnicity=1 ○ Yes
- Ethnicity=2 ○ No
- Ethnicity=3 ○ Prefer not to say

Educ

What is the highest level of education you have completed?

- Educ=1 ○ 8th grade or less
- Educ=2 ○ Some high school
- Educ=3 ○ High school graduate or equivalent
- Educ=4 ○ Some college
- Educ=5 ○ Associate's or Bachelor's degree

© 2021 Gale R et al. *JAMA Network Open.*

Educc=6 Master's degree or higher

Marital

Are you now married, living with a partner, widowed, divorced, separated, or never married?

- Marital=1 Married
- Marital=2 Living with a partner
- Marital=3 Widowed
- Marital=4 Divorced
- Marital=5 Separated
- Marital=6 Never married

© 2021 Gale R et al. *JAMA Network Open.*

0% 100%

income

What is the best estimate of your household’s total annual income?

- income=1 ○ Less than $50,000
- income=2 ○ Between $50,000 to $100,000
- income=3 ○ Between $100,001 to $200,000
- income=4 ○ More than $200,000
- income=5 ○ Prefer not to say

Employment

Which of the following categories best describes your employment status? (select all that apply)

- Employment_1 ☐ Homemaker
- Employment_2 ☐ Unemployed
- Employment_3 ☐ Retired
- Employment_4 ☐ On disability
- Employment_5 ☐ On leave of absence from work
- Employment_6 ☐ Full-time worker (40 or more hours per week)
- Employment_7 ☐ Part-time worker (less than 40 hours per week)
- Employment_8 ☐ Full-time student
- Employment_9 ☐ Part-time student

InsurancePlan

Do you currently have health insurance or health coverage?

© 2021 Gale R et al. *JAMA Network Open.*

InsurancePlan=1

Yes

InsurancePlan=2

No

usoc

Is there a place that you usually go to when you are sick or need advice about your health?

usoc=1

Yes

usoc=2

No

© 2021 Gale R et al. *JAMA Network Open.*

0% 100%

usocloc

What kind of place do you go to most often when you are sick or need advice about your health?

usocloc=1 ○ Doctor's office

usocloc=2 ○ Clinic, health center, or hospital clinic

usocloc=3 ○ Urgent care

usocloc=4 ○ Emergency room

usocloc=5 ○ No one place

usocloc=6 ○ Other place(s)

usocloc=7 ○ I don't know

→

© 2021 Gale R et al. *JAMA Network Open.*

0% 100%

Tobacco

Altogether, have you smoked at least 100 or more cigarettes in your entire lifetime?

- Tobacco=1 Yes
- Tobacco=2 No

Tobacco2

Do you now smoke cigarettes every day, some days, or not at all?

- Tobacco2=1 Every day
- Tobacco2=2 Some days
- Tobacco2=3 Not at all

Drinking

On average, how many days out of the week do you have at least one drink of any alcoholic beverage such as beer, wine, a malt beverage, or liquor?

- Drinking=1 None at all
- Drinking=2 1 to 3 days
- Drinking=3 4 to 6 days
- Drinking=4 Every day of the week

© 2021 Gale R et al. *JAMA Network Open.*

0% 100%

HHoldSize

How many people live in your household, including yourself, children, and adults? [ ]

→

© 2021 Gale R et al. *JAMA Network Open*.

0% 100%

hhhighrisk

Is anyone in your household at higher risk for severe illness from COVID-19 if they were to catch it (eg, age 65 years and older, immunocompromised, has a serious underlying medical condition)?

hhhighrisk=1 Yes

hhhighrisk=2 No

© 2021 Gale R et al. *JAMA Network Open.*

0% 100%

Dependents1

How many children or dependents live in your household?

0% 100%

HealthQuality

In general, would you say your health is: excellent, very good, good, fair, or poor?

- HealthQuality=1 Excellent
- HealthQuality=2 Very good
- HealthQuality=3 Good
- HealthQuality=4 Fair
- HealthQuality=5 Poor

→

© 2021 Gale R et al. *JAMA Network Open.*

0% 100%

ComorbidityQ

The following is a list of common problems. Please indicate if you currently have the problem.

	Do you have the problem?	
**Problem**	**No**	**Yes**
Heart disease	ComorbidityQ_r1=1 ◯	ComorbidityQ_r1=2 ◯
High blood pressure	ComorbidityQ_r2=1 ◯	ComorbidityQ_r2=2 ◯
Lung disease	ComorbidityQ_r3=1 ◯	ComorbidityQ_r3=2 ◯
Diabetes	ComorbidityQ_r4=1 ◯	ComorbidityQ_r4=2 ◯
Ulcer or stomach disease	ComorbidityQ_r5=1 ◯	ComorbidityQ_r5=2 ◯
Kidney disease	ComorbidityQ_r6=1 ◯	ComorbidityQ_r6=2 ◯
Liver disease	ComorbidityQ_r7=1 ◯	ComorbidityQ_r7=2 ◯
Anemia or other blood disease	ComorbidityQ_r8=1 ◯	ComorbidityQ_r8=2 ◯
Cancer	ComorbidityQ_r9=1 ◯	ComorbidityQ_r9=2 ◯
Depression	ComorbidityQ_r10=1 ◯	ComorbidityQ_r10=2 ◯
Osteoarthritis, degenerative arthritis	ComorbidityQ_r11=1 ◯	ComorbidityQ_r11=2 ◯
Back pain	ComorbidityQ_r12=1 ◯	ComorbidityQ_r12=2 ◯

© 2021 Gale R et al. *JAMA Network Open.*

Rheumatoid arthritis	ComorbidityQ_r13=1 ◯	ComorbidityQ_r13=2 ◯
Other medical problems	ComorbidityQ_r14=1 ◯	ComorbidityQ_r14=2 ◯

→

© 2021 Gale R et al. *JAMA Network Open.*

0% 100%

Religion

Which of the following, if any, best describes your religion? (select all that apply)

- Religion_1 ☐ Agnostic
- Religion_2 ☐ Atheist
- Religion_3 ☐ Buddhist
- Religion_4 ☐ Christian (Catholic, Protestant, or any other Christian denomination)
- Religion_5 ☐ Hindu
- Religion_6 ☐ Jewish
- Religion_7 ☐ Muslim
- Religion_8 ☐ Other religion
- Religion_9 ☐ None of the above
- Religion_10 ☐ Prefer not to say

→

© 2021 Gale R et al. *JAMA Network Open.*

0% 100%

politicalparty

Generally speaking, do you usually think of yourself as a Democrat, an Independent, a Republican, or something else.

- politicalparty=1 ◯ Democrat
- politicalparty=2 ◯ Independent
- politicalparty=3 ◯ Republican
- politicalparty=4 ◯ Something else
- politicalparty=5 ◯ Prefer not to say

→

© 2021 Gale R et al. *JAMA Network Open.*

0% 100%

News1

Where do you most frequently receive your news?

- News1=1 ◯ National or cable TV news (eg, ABC, CBS, CNN, Fox News, MSNBC, NBC)
- News1=2 ◯ Local TV news
- News1=3 ◯ Newspaper
- News1=4 ◯ Online news websites
- News1=5 ◯ Social media websites
- News1=6 ◯ Other source
- News1=7 ◯ I do not read, watch, or listen to the news

→

© 2021 Gale R et al. *JAMA Network Open.*

0% 100%

News2

You mentioned that you receive your news through national or cable TV news. Which channel do you normally watch?

- News2=1 ○ ABC
- News2=2 ○ CBS
- News2=3 ○ CNN
- News2=4 ○ Fox News
- News2=5 ○ MSNBC
- News2=6 ○ NBC
- News2=7 ○ Other channel
- News2=8 ○ Prefer not to say

→

© 2021 Gale R et al. *JAMA Network Open.*

0% 100%

USStateNames

Which state do you live in?

© 2021 Gale R et al. *JAMA Network Open*.

eAppendix 2. Myocardial Infarction Scenario Descriptions

These descriptions of the scenario and its attributes were shown to participants prior to the myocardial infarction conjoint exercise to facilitate their understanding of each characteristic. COVID-19; Coronavirus Disease 2019; ER, emergency room.

Experiencing Symptoms Likely Related to a Heart Attack

For the next few pages, imagine that you are experiencing chest pain that came on suddenly. The pain is likely related to a **heart attack and the risk of dying from it without treatment is 40%**. Receiving treatment in the ER will reduce your risk of dying from a heart attack. However, going to the ER may potentially expose you to others who have COVID-19.

Below are three considerations that may affect your decision about whether or not to visit the ER. Please read the information carefully as it will help as you go through the next exercise.

Reduction in Chance of Dying from Heart Attack because of ER Treatment
This is how much your chance of dying from a heart attack goes down if you receive treatment in the ER.

Chance of Catching COVID-19 in the ER
This is the chance of catching COVID-19 in the ER from other patients, doctors, nurses, or other members of the medical team.

Crowdedness of ER with Other Patients
This represents how crowded the ER is with other patients who are seeking care.

© 2021 Gale R et al. *JAMA Network Open.*

eAppendix 3. Appendicitis Scenario Descriptions

These descriptions of the scenario and its attributes were shown to participants prior to the appendicitis conjoint exercise to facilitate their understanding of each characteristic. COVID-19; Coronavirus Disease 2019; ER, emergency room.

Experiencing Symptoms Likely Related to Appendicitis

For the next few pages, imagine that you are experiencing belly pain that came on suddenly. The pain is likely related to **appendicitis (ie, inflammation of the appendix) and the risk of dying from it without treatment is 4%.** Receiving treatment in the ER will reduce your risk of dying from appendicitis. However, going to the ER may potentially expose you to others who have COVID-19.

Below are three considerations that may affect your decision about whether or not to visit the ER. Please read the information carefully as it will help as you go through the next exercise.

Reduction in Chance of Dying from Appendicitis because of ER Treatment
This is how much your chance of dying from appendicitis goes down if you receive treatment in the ER.

Chance of Catching COVID-19 in the ER
This is the chance of catching COVID-19 in the ER from other patients, doctors, nurses, or other members of the medical team.

Crowdedness of ER with Other Patients
This represents how crowded the ER is with other patients who are seeking care.

© 2021 Gale R et al. *JAMA Network Open.*

eTable 1. Demographic Comparisons Between 1517 Survey Noncompleters and Completers

Variable	Survey non-completers (n=584)	Survey completers (n=933)	*P*
Age (years), mean (SD)[a]	33.4 (16.9)	40.1 (13.0)	<.001
Sex, n (%)[b]:			<.001
Male	214 (46.2)	438 (47.0)	
Female	222 (48.0)	491 (52.6)	
Prefer not to say	27 (5.8)	4 (0.4)	

The unpaired Student t-test and chi-squared test were used to compare continuous parametric and categorical data, respectively.

a Data are missing from 118 individuals among the survey non-completers.

b Data are missing from 121 individuals among the survey non-completers.

© 2021 Gale R et al. *JAMA Network Open.*

eTable 2. Demographics of Study Cohort vs US Population

Variable	Study cohort (N=933) n (%)	2019 US Population % (latest available data)
Age:		
18-29 yo	230 (24.7%)	21.0%
30-39 yo	232 (24.9%)	17.3%
40-49 yo	218 (23.4%)	15.9%
50-59 yo	165 (17.7%)	16.4%
≥60 yo	88 (9.4%)	29.4%
Sex:		
Male	438 (47.0%)	48.7%
Female	491 (52.6%)	51.3%
Prefer not to say	4 (0.4%)	---
Race/ethnicity:		
Non-Hispanic White	594 (63.7%)	59.5%
Non-Hispanic Black	109 (11.7%)	11.8%
Latino	109 (11.7%)	15.5%
Non-Hispanic Asian	71 (7.6%)	5.6%
Other	50 (5.4%)	7.6%
Education level[a]:		
High school degree or less	247 (26.5%)	38.3%
Some college	205 (22.0%)	20.0%
College degree	355 (38.1%)	29.0%
Graduate degree	126 (13.5%)	12.8%
Marital status[b]:		
Married	361 (38.7%)	47.6%
Not married	572 (61.3%)	52.4%
Total household annual income:		
≤$50,000	410 (43.9%)	38.4%
$50,001–$100,000	310 (33.2%)	30.2%
≥$100,001–$200,000	156 (16.7%)	31.4%
Prefer not to say	57 (6.1%)	---
Employment status[c]:		
Unemployed, on disability, on leave of absence from work, retired, or homemaker	365 (39.1%)	36.4%
Employed or student	568 (60.9%)	63.6%
US region:		
Northeast	179 (19.2%)	17.4%
South	348 (37.3%)	38.1%
Midwest	205 (22.0%)	20.8%
West	201 (21.5%)	23.8%
Insurance status[d]:		
Insured	775 (83.1%)	89.7%
Not insured	158 (16.9%)	10.3%

US population data estimates are from the 2019 US Census American Community Survey (https://data.census.gov/). Data were accessed on May 31, 2021.

a The US population data are calculated among those ≥25 years old.
b The US population data are calculated among those ≥15 years old.
c For the US population data, we report the proportion of the population ≥16 years old that is in the labor force.
d The US population data are calculated among those ≥19 years old.

© 2021 Gale R et al. *JAMA Network Open.*

eTable 3. Regression Analyses on Prioritizing Avoidance of COVID-19 Exposure With More Restrictive *P* Value Threshold

Variable	Prioritized avoidance of COVID-19 exposure in the ED	
	**Myocardial infarction scenario aOR [95% CI]**	**Appendicitis scenario aOR [95% CI]**
Age:		
18-29 yo	Reference	Reference
30-39 yo	1.56 [0.89-2.74]	1.25 [0.79-1.99]
40-49 yo	1.99 [1.11-3.56]	1.08 [0.66-1.75]
50-59 yo	1.42 [0.74-2.71]	0.76 [0.44-1.31]
≥60 yo	0.65 [0.24-1.79]	0.53 [0.25-1.15]
Race/ethnicity:		
Non-Hispanic White	Reference	Reference
Non-Hispanic Black	1.56 [0.87-2.80]	1.55 [0.94-2.57]
Latino	0.75 [0.36-1.54]	0.83 [0.47-1.47]
Non-Hispanic Asian	1.39 [0.67-2.92]	1.42 [0.76-2.66]
Other	1.16 [0.51-2.63]	1.34 [0.66-2.75]
Educational attainment:		
High school degree or less	Reference	Reference
Some college education	0.78 [0.45-1.34]	1.21 [0.76-1.91]
College degree	0.74 [0.44-1.24]	0.78 [0.50-1.22]
Graduate degree	0.90 [0.43-1.85]	0.88 [0.47-1.64]
Total household income:		
<$50,000	Reference	Reference
$50,000–$100,000	0.80 [0.50-1.28]	0.99 [0.66-1.46]
≥$100,001	0.51 [0.24-1.05]	0.70 [0.39-1.24]
Prefer not to say	2.51 [1.22-5.19]	2.11 [1.09-4.06]
Current smoking status:		
Not at all	Reference	Reference
Some days	2.42 [1.29-4.56]	1.48 [0.83-2.65]
Every day	0.83 [0.49-1.41]	1.01 [0.65-1.58]
Has health insurance	1.46 [0.87-2.47]	0.80 [0.52-1.23]
Has usual source of care	0.50 [0.33-0.76]	0.58 [0.40-0.83]
Religion:		
Buddhist, Christian, Hindu, Jewish, Muslim, or other religion	Reference	Reference
Agnostic or atheist	1.12 [0.57-2.18]	0.88 [0.49-1.57]
None of the above	1.51 [0.93-2.45]	1.16 [0.76-1.79]

© 2021 Gale R et al. *JAMA Network Open.*

Prefer not to say	0.42 [0.14-1.23]	0.67 [0.28-1.60]
Political party affiliation:		
Democrat	Reference	Reference
Independent	0.82 [0.49-1.36]	1.14 [0.75-1.74]
Republican	0.65 [0.37-1.13]	0.72 [0.45-1.15]
Something else	1.20 [0.50-2.84]	0.58 [0.24-1.39]
Prefer not to say	0.88 [0.35-2.23]	1.16 [0.54-2.51]
Main source of news:		
National or cable television news	Reference	Reference
Local television news	1.33 [0.77-2.29]	1.19 [0.75-1.88]
Newspaper	1.06 [0.25-4.48]	0.33 [0.07-1.62]
Online news websites	0.70 [0.35-1.41]	0.83 [0.48-1.43]
Social media websites	1.14 [0.62-2.10]	1.04 [0.63-1.73]
Other source	1.91 [0.71-5.16]	2.03 [0.83-4.97]
Do not read, watch, or listen to the news	3.24 [1.57-6.65]	1.87 [0.96-3.62]
Perceived chance of contracting COVID-19:		
1% or less	Reference	Reference
2%	0.46 [0.23-0.92]	0.54 [0.31-0.95]
4%	0.29 [0.13-0.62]	0.44 [0.24-0.78]
6%	0.65 [0.33-1.32]	0.60 [0.33-1.10]
8%	0.45 [0.18-1.11]	0.37 [0.17-0.79]
10% or more	0.58 [0.36-0.95]	0.56 [0.37-0.86]
Wearing a face mask in public is an important step for protecting other people and slowing the spread of COVID-19:		
Strongly disagree	Reference	Reference
Disagree	0.60 [0.18-2.00]	0.57 [0.20-1.63]
Neither agree or disagree	1.60 [0.72-3.55]	1.86 [0.91-3.80]
Agree	0.53 [0.25-1.15]	0.68 [0.34-1.33]
Strongly agree	0.58 [0.28-1.21]	0.76 [0.40-1.44]

aOR, adjusted odds ratio; CI, confidence interval; COVID-19, Coronavirus Disease 2019; ED, emergency department. The multivariable logistic regression models included all covariates in the table; these variables were selected for inclusion in the models as they had $p<.01$ in bivariate analyses.

© 2021 Gale R et al. *JAMA Network Open.*
